# Group meaningfulness and the causal direction of influence between the ingroup and the self or another individual: Evidence from the Induction-Deduction Paradigm

**DOI:** 10.1371/journal.pone.0229321

**Published:** 2020-03-10

**Authors:** Mara Cadinu, Andrea Carnaghi, Francesca Guizzo

**Affiliations:** 1 DPSS, University of Padova, Padova, Italy; 2 DSV, University of Trieste, Trieste, Italy; Sapienza University of Rome, ITALY

## Abstract

The goal of the present study was to investigate the causal direction of influence between the ingroup as a whole and the self or another ingroup member considering a key feature of groups, i.e., their perceived meaningfulness. To this goal, in Study 1, 2, and 3 we predicted a preference for self-stereotyping and ingroup-stereotyping in the meaningful social categories of sorority women, left-handed people and psychology students. In Study 4 we further expect that the meaningfulness attributed to a group moderates the direction of causality between individual and ingroup perception. Thus, we used one’s Zodiac sign as the ingroup whose degree of meaningfulness varies across participants and we hypothesized higher levels of meaningfulness attributed to the ingroup to be associated with higher self- and ingroup-stereotyping. Using the methodologically stringent Induction Deduction Paradigm, participants were given information on unfamiliar dimensions, about either the ingroup or an individual (self or other ingroup member) and asked to make inferences on those same attributes about the ingroup (induction condition) or the individual (deduction condition). As predicted, a preference for deduction to the self (i.e., self-stereotyping) and deduction to another ingroup member (i.e., ingroup-stereotyping) were found for the meaningful groups of sorority women, left-handed people, and Psychology students (Studies 1, 2, and 3). In Study 4, consistent with predictions, the higher the level of attributed meaningfulness to the Zodiac system the higher the degree of deduction both to the self (self-stereotyping) and to another Zodiac ingroup member (ingroup-stereotyping). Several implications of these results are discussed, for example in relation to the possibility of educational interventions aimed at invalidating intergroup differences.

## Introduction

Social observers are active meaning-makers, namely they seek and build up coherent and unified relations among events [1]. In doing so, the social environment ends up comprising expected relations among its constituents, and these expected relations are processed as being *meaningful* given that they *signal an assumed reality* [1]. Based on this theoretical perspective, we define a group as *meaningful* when it is perceived as psychologically real. At the empirical level, the issue of meaningfulness has been studied with respect to stereotyping in general (e.g., [2]) and within the entitativity framework specifically (e.g., [3,4]). However, no study so far has addressed the causal relation between the representation of the self and the representation of the ingroup in meaningful group contexts, namely whether individuals derive their representation from the representation of the ingroup or viceversa in meaningful group contexts. In this regard, previous studies have addressed the causality issue in existing groups, but they did not focus on the specific issue of group meaningfulness. Moreover, these studies relied on paradigms that do not allow for a stringent test of the direction of causality involved in the self-ingroup representation (e.g., [5–7]). By contrast, the causal direction of inference involving the representation of the self and the ingroup has been firmly established in minimal group settings, which, by virtue of their nature, are not meaningful groups [8]. Hence, the present research program aims to fulfill this lacuna by analyzing for the first time the role of group meaningfulness in the causal relation between the representation of the self and the representation of the ingroup as a whole by adopting a methodologically rigorous paradigm (i.e. Induction Deduction Paradigm; [8]).

In line with research showing that perceivers take advantage of group stereotyping in the construal of group members when the group is meaningful (e.g., [2,9,10]), we reasoned that in such contexts the representation of the individual will derive from ingroup representation (i.e., deduction or stereotyping) to a greater extent than the representation of the ingroup stems from the representation of the individual ingroup member (i.e., induction or anchoring). Importantly, we further expect that the attributed meaningfulness to a group moderates the direction of causality between individual and ingroup perception. Specifically, we expect the above-mentioned pattern of results to occur when the ingroup is perceived as meaningful, whereas we expect participants to derive the representation of the ingroup from the representation of the self when the ingroup is perceived as meaningless, thus mimicking the pattern of findings reported in minimal group contexts [8]. We argue that this pattern follows the rules of logic that in meaningful groups the group represents a more reliable source of information to base the inference process than an individual group member (self or another group member), whereas in less meaningful groups the group is not a reliable source of information and participants anchor their inference process on the self, which represents the only reliable source available.

### Meaningfulness in group representation

Once established, *meaningful* representations allow observers to understand, predict and control their social environment because they ‘account for cause and effect relations, as well as teleological relations […]’ ([1], p. 96).

Social observers are highly sensitive as to whether the relation between a category and its instances is a meaningful relation [1]. Indeed, meaningful social categories, but not meaningless social categories, signal expected differences between categories and are construed as diagnostic of category members’ characteristics, behaviors, and traits [2,11,4]. Also, group meaningfulness, as an aspect of group entitativity, together with group coherence (e.g., [3]), has been shown to be positively correlated with group stereotyping [10]. Although these studies did not explicitly address the direction of causality between the perception of the ingroup and the individual (self or other ingroup member), they help extend the definition of group meaningfulness to imply not only psychological reality but also a similarity relation between the group and its individual members. As additional factors may account for the perceived similarity relation between the group and its individual members, it is important to clarify that group meaningfulness differs from these psychological factors that are relevant to the group representation as well as to the representation of its individual members. Among them is ingroup identification as a whole, which refers to the degree to which one feels attached and considers the group important to the self and to one’s definition. One may consider the ingroup of Psychology students as meaningful, but neither identify with nor consider oneself as typical of this group. This argument is in line with accumulated evidence demonstrating that ingroup identification and ingroup meaningfulness, together with other components of ingroup entitativity, are two distinct constructs (see [12], p. 139–140). Second, given that self-typicality, i.e. perceived prototypicality of the self with respect to the ingroup, is considered a component of ingroup identification [13], it should also be considered as separate from meaningfulness. Third, group meaningfulness differs from group clarity. The clarity of a group representation (i.e., social category) refers to the extent to which its content is well or poorly defined [14]. Research addressing the role of group clarity ([14]; see also, [15,16]) focused on the antecedents of in-group identification, being this self-anchoring for unclear ingroup representation and self-stereotyping for clear ingroup representation. Importantly, group clarity is not a synonym of group meaningfulness. Indeed, perceivers may hold a clear or an unclear prototype of the Libra sign as a category. However, regardless of having a clear *knowledge* of group representation, perceivers may not consider the Zodiac system as meaningful because it is not able to establish an expected relation between *Libra* and its members’ personality. In other words, knowledge about the *Libra* content may be clear but this content may be meaningless to perceivers.

In sum, given the theoretical and psychological importance of the construct of group meaningfulness, considering that this construct is not confounded with neighbor concepts, and since no study has thus far addressed the individual-ingroup dynamics in the context of meaningful groups, the current set of studies fulfills this theoretical gap by analyzing, and comparing, the relative strength of the direction of causality between the individual and the ingroup representation in meaningful group contexts.

### The direction of causality between the individual and the group

So far, the role of meaningfulness has not been investigated in the assessment of the direction of causality between the individual and the group. However, some studies have compared the strength of self-anchoring and self-stereotyping in existing groups, which we interpret as typically highly meaningful groups. Guimond et al. [6] proposed a prevalence of self-stereotyping versus self-anchoring, Otten and Epstude [7] proposed a prevalence of self-anchoring versus self-stereotyping, whereas Cho and Knowles ([5]; Study 4) proposed an equal contribution of self-anchoring and self-stereotyping toward self-ingroup similarity. Notwithstanding the importance of these studies in the area of self-ingroup dynamics, they are all based on paradigms that do not allow to stringently test the causal direction of inference between the self and the ingroup for two important reasons. First, they do not experimentally manipulate self-perception and ingroup perception, but ask participants to report their view on the self and the ingroup on a series of traits. Second, they do not control for participants’ previous knowledge on the self or the ingroup, as they relied on familiar dimensions (e.g., to be nice) that can be well acknowledged as a part of the self or/and the ingroup representation. Therefore, no conclusions can be drawn on the causal direction between self and ingroup perception in existing groups. Most important, in these studies group meaningfulness, which is central to our study, was not directly investigated.

As opposed to existing groups, the causal direction between self and ingroup perception has been firmly established in research based on minimal groups [8,17–21]. Cadinu and Rothbart employed the so-called Induction Deduction Paradigm and showed a clear preference to rely on induction (i.e., self-anchoring) than deduction (i.e., self-stereotyping) when the self is involved, and a preference to rely on deduction (i.e., ingroup-stereotyping) than induction (i.e., other-anchoring) when another ingroup member is involved.

In the current research program, we recast the Induction Deduction Paradigm, which has only been used with minimal groups thus far, within the context of meaningful groups. Importantly, Induction Deduction Paradigm allows us to overcome limitations of previous research with existing groups. Specifically, because the relationship between self and ingroup perception is complicated by the fact that information about both representations is typically available, the Induction Deduction Paradigm allows the researcher to experimentally manipulate feedback provided to participants about either the ingroup or the individual along *unfamiliar* or fictitious dimensions, thus allowing to directly test the direction of causality between self and ingroup representation. Moreover, the present paradigm extends previous research by investigating the relation between the ingroup and an ingroup member other than the self, a feature that has both methodological and theoretical advantages. First, testing the inferential pattern between the individual and the ingroup when the self is not involved allows researchers to assess the effects of type of inference alone (induction vs. deduction). Second, this test would help clarify whether self-stereotyping and ingroup-stereotyping [22] stem from a similar inferential process. Third, we would be able to compare results under low meaningfulness to findings with minimal groups by Cadinu and Rothbart [8]. Hence, we employed the Induction Deduction Paradigm to test the degree of inference between individual and ingroup in the context of meaningful groups.

### Hypotheses

The core prediction of the present study is that in meaningful groups (Studies 1, 2, and 3) the similarity between the self and the ingroup is ruled by a deductive (i.e., self-stereotyping) rather than inductive (i.e., self-anchoring) inference process, in line with studies attesting that meaningful categories are construed as diagnostic of category members’ characteristics (e.g., [2]). Also, and as for meaningful groups, in line with the tendency to perceive the self and other ingroup members in a similar way (i.e., depersonalization; see Self-Categorization Theory, [23]), we predict a similar deductive inference process for another ingroup member, whose representation should also be derived from ingroup representation, i.e., ingroup-stereotyping (e.g., [22]). This predicted preference for deduction over induction is based on the theoretical premise of the present study that a meaningful group signals expected relations among the category and its members so that characteristics of the group can be generalized to both the self and another ingroup member (e.g., [1,11]). Specifically, we expect participants to prefer deduction over induction because they would apply the rules of logic that it is more reliable to base inferences on the average of multiple data points (i.e., information on a meaningful ingroup) and then generalize group average information to the individual (i.e., self-stereotyping and ingroup-stereotyping) than to generalize a single data-point on one individual (self or other ingroup member) to the whole group (i.e., self-anchoring and other-anchoring).

To test more stringently the core prediction of the present study, we provide an experimental test of the hypothesis that the degree of deduction (self-stereotyping and ingroup-stereotyping) is moderated by the perceived meaningfulness of the ingroup (Study 4). The same ingroup can be perceived, at least in certain cases, as more or less psychologically real depending on its meaningfulness to the perceiver. When the ingroup is perceived as meaningful, we expect to obtain results similar to the expected findings of Studies 1, 2, and 3, with the deductive process stronger than the inductive process both when the self or another ingroup member is the target. By contrast, when the ingroup is perceived as a non-meaningful group, we predict a preference for induction over deduction when the self is involved (i.e., self-anchoring), but a preference for deduction over induction when another ingroup member is the target (i.e., ingroup-stereotyping), thus mimicking previous results obtained in minimal group contexts by Cadinu and Rothbart ([8]; see also, [24]). It should be noticed that the expected prevalence of induction over deduction in non-meaningful groups pertains only to the self but not to the other ingroup member. The reason for this prediction is that in non-meaningful groups participants do not possess reliable information about the ingroup to base their inferences, but they possess reliable information on the self that can therefore be generalized to the ingroup, thus resulting in a preference for self-anchoring. Still in the context of non-meaningful groups, more difficult is the condition in which another ingroup member is involved in the inference task. In this case, because no reliable information is present either on the ingroup or another ingroup member, participants would follow the rules of logic prescribing that it is more reliable to base inferences on group average than to generalize a single data-point on one individual to the whole group, thus resulting in a preference for ingroup-stereotyping.

In addition, given the importance of ingroup identification and self-typicality in previous studies on self-stereotyping (e.g., [13,25]), in the present Studies 1, 2, and 3 we assessed participants’ self-typicality and ingroup identification to explore as to whether these variables might be potential moderators of the inference process in meaningful group settings.

In the following studies, we report all measures, manipulations and exclusions.

## Study 1

The group addressed is Sorority women, a group relatively small in size (between about 40 and 80 members), in which membership is determined by mutual preferences of members and Sorority house. Importantly, sorority women were chosen as the meaningful group for Study 1 based on previous research showing that Greek system groups are perceived as high in entitativity [3], which includes perceived meaningfulness [10]. We hypothesize stronger preference to rely on deduction than induction both when the self and another individual are involved in the inference task.

### Method

#### Participants

The number of participants was a priori determined based on a study using the same experimental design ([8], Exp. 4, *N* = 153, *f* = .23). A priori power analysis based on Cadinu and Rothbart’s effect size *f* = .23, α = .05, and Power 1 –β = .80 indicated a required total sample size of *N* = 151 participants. Given practical constraints to recruit participants (e.g., need to limit the number of sororities involved) the experimental sample approached the required *N* but was lower than expected. The final sample included *N* = 123 sorority women from two different sorority houses (*n* = 76, *n* = 47) who volunteered to participate in this experiment and were given 5$ for their participation. The sensitivity power analyses (α = .05, 1 - β = .80) computed on the available experimental sample (*N* = 123) showed that the minimal detectable effect (MDE) Cohen’s *f* = .25 was very similar to the size of the effect reported by Cadinu and Rothbart [8]. Moreover, the sensitivity analyses indicated that the smallest effect size we were able to detect with the present sample size fell in the small effect area [26].

All the studies were carried out in accordance with the recommendations of APA ethical guidelines and received the approval of the University of Oregon IRB. All participants gave written informed consent in accordance with the Declaration of Helsinki.

#### Design and procedure

The experiment was conducted at the participants’ sorority house. Following the Induction Deduction Paradigm procedure [8], participants were asked to perform individually four “cognitive tasks” involving attention, memory, and mental assembling skills. They were given instructions for each task separately with no mention of the purpose of any task, and no suggestion that feedback would be provided later. After the four tasks, explained below, the experimenter left the room for five minutes, allegedly to score participants' performance. When the experimenter returned to the room, she gave each participant a packet for the next task. Using a 2 (Type of Inference: induction vs. deduction) X 2 (Type of Individual: self vs. other) between participants design, participants were randomly assigned to either an induction condition, in which they received information about how they (*n* = 30) or another person belonging to their sorority house (*n* = 32) scored on previous tasks and were asked to make inferences about people belonging to their sorority house as a whole, or a deduction condition, in which participants received information about how people belonging to their sorority house as a whole scored on previous tasks and were asked to make inferences about the self (*n* = 31) or another sorority house’s member (*n* = 30). Information was provided in the form of scores (i.e., 2, 4, 6 or 8) along eight favorable but unfamiliar psychological dimensions for a total of eight judgments. Scores were randomized across traits for each participant. After looking at each marked scale, all participants had to judge the degree to which the same dimension was characteristic or uncharacteristic of the inference target for a total of eight judgments. Here is an example of instructions provided to participants in the deduction-to-other (ingroup-stereotyping) condition:

On each of the following four pages, you will be given information about four psychological characteristics derived from the tasks that you performed earlier. On each page, you will first receive the score that the people belonging to your sorority house as a whole received on that measure. Underneath that information, we would like you to estimate, as best as you can, how an individual member of your sorority house might have scored on the same measure. The single individual that you are being asked about was in this experiment on a previous day, but at the same time as yourself.

*Stimulus materials used for the cognitive tasks*. The materials (see S1 Material and S2 Material) for the four cognitive tasks were presented by slides. For the first task, participants saw a series of slides containing a pattern of seven large letters (two A’s, one C, two Ds, one E, one F), each of them made of small alphabet letters (for example, a big A could be made of small Ds). Participants were then asked questions like “How many times did you see a big A?”, “How many times did you see a letter composed of small A's?”. For the second task, participants were presented with a slide showing a list of color words (for example, "black," "red," and "green") written in different colors. Participants were then asked questions like: *How many words were written in red*? *How many times did the word "red" appear (regardless of the color it was written in)*? For the third task, participants were presented with a slide showing a list of 15 nouns, five of which were animals, five trees, and five tools. At the end, participants were asked to write down as many of the words from the list as they could recall. For the fourth and final task, participants were presented with slides containing 15 visual shapes (e.g., a sphere, a wire, a cross, a flat square). For each of the three trials that made up the fourth task, the experimenter named three shapes and asked participants to mentally assemble them to make an interesting and potentially useful object. For each trial, participants had to close their eyes for 1 min to mentally assemble the parts and then draw the object without being allowed to look at the slide until they were done.

*Stimulus materials for the inference task*. Participants were presented with eight 9-point scales measuring the following fictitious psychological dimensions: *Parallel Information Processing*, *Clustering in Semantic Recall*, *Global Orientation in Pattern Construction*, *Modality Dominance in Synesthesic perception*, *Lateralization of brain functions in Mental Assembling*, *Image Orientation in Constructive Skills*, *Discriminatory Perception in Serial Cognition*, and *Analytic Representation in Inhibitory Tasks*. Each scale indicated a favorable dimension, the meaning of which was explained. For example, participants read that high scores on the *Clustering in Semantic Recall* scale indicate good performance in recall and recognition tasks. As a consequence, low scores indicate unfavorable performance. In order to counterbalance the association between scores and dimensions, we provided participants with scores that varied from low to high (i.e., 2, 4, 6 or 8), which could be associated with any dimension in a counterbalanced order. After looking at each marked scale indicating a score for the anchor (ingroup or individual) participants had to judge the degree to which the same dimension was characteristic of the target (individual or ingroup as a whole) using an identical scale. For each scale, participants were also asked to rate its social desirability on a 9-point scale that ranged from *very undesirable* (1) to *very desirable* (9). See S3 Material.

*Self-typicality*. At the very end participants were asked how typical they were of their sorority house on a scale ranging from 1 = *Not at all typical* to 9 = *Very typical*.

### Results

#### Inferred similarity between group and individual

A between-persons design was used. For each participant, we computed an average difference score (i.e., average d^2^) representing the average of squared differences between scores provided to participants and inferred scores. Thus, lower scores indicate stronger inferred similarity between ingroup and individual. Preliminary analyses were performed on average within-person d^2^ scores to exclude the moderating role of the Type of Sorority (Sorority A vs. Sorority B) involved in the study. We also included Type of Inference (induction or deduction) and Type of Individual (self or other) as (between-persons) factors. Type of Sorority produced only a main effect (*F*(1,115) = 4.83, *p* = .03, η^2^ = .04) but, importantly, did not interact with any other variable. Therefore, Type of Sorority was not a significant moderator. We then perform a 2 X 2 ANOVA on average within-person d^2^ scores with Type of Inference (induction or deduction) as the first (between-persons) factor and Type of Individual (self or other) as the second (between-persons) factor controlling for Type of Sorority. As predicted, a marginally significant effect of Type of Inference was found (*F*(1,118) = 3.81, *p* = .053, η^2^ = .03), with a general preference to rely on deduction than induction (see Table 1). As predicted, no other effects were found (*ps* > .24). Finally, when the average level of self-typicality with the ingroup was included in the general linear model, neither a main effect of self-typicality (*p* > .42) nor any interaction with other variables was found (*ps* > .27). Therefore we included self-typicality as a covariate in the ANOVA and the effect of Type of Inference was still marginally significant (*F*(1,118) = 3.83, *p* = .053, η^2^ = .03). No other effects were found (*p >* .32*)*.

**Table 1:** Study 1, Study 2, and Study 3. Average within-person d^2^ scores as a function of Type of Inference (induction vs. deduction) and Number of Study. Standard deviations are in parenthesis.

	Induction *M* (*SD*)	Deduction *M* (*SD*)	η^2^
Study 1 (Sorority)	4.64 (3.23)_†_	3.62 (2.82)_†_	0.03
Study 2 (Left-handed)	5.00 (2.76)_a_	3.24 (3.06)_b_	0.11
Study 3 (Psychology students)	4.73 (2.21)_a_	3.24 (2.44)_b_	0.09

Means across each row that do not share the same subscript are significantly different from each other at p < .05, and _†_equals to p = .053.

Overall, participants tended to rely on deduction versus induction and this effect was independent of Type of Individual (self vs. other) and self-typicality.

## Study 2

The goal of Study 2 is to strengthen and extend Study 1 findings to a different type of meaningful group, namely left-handed people. Unlike sorority, membership in the left-handed group is not the result of mutual choice by members and group, but is determined by genetic characteristics, thus providing a good arena to test the generalizability of results from Study 1 to a different type of meaningful ingroup. It is worth noting that we conducted a pretest to demonstrate that left-handed people perceive the ingroup as a meaningful group.

### Method

#### Pre-test

To assess the meaningfulness of the group of left-handed people we used an on-line survey (*N* = 49). The meaningfulness scale included the following instructions. *Some groups are more “groups” than others*. *Use the following scales to express your opinion*: *Left-handed people can be defined as a group*, *Left-handed people feel part of the left-handed people group*, *It makes sense to consider left-handed people as a real group*, *Left-handed people are a meaningful group*. The first two items were taken from the entitativity scale by Spencer-Rodgers et al. [10]. Responses ranged from 1 (*Not at all*) to 7 (*A lot*). Participants also rated other presumably non-meaningful groups by using the same scale (see Table 2). An average meaningfulness score was calculated for each group (all αs were above .80). Left-handed individuals rated their ingroup more meaningful than the other non-meaningful groups (see Table 2). Moreover, as predicted, left-handed individuals perceived their ingroup as significantly *more* meaningful than the midpoint of the scale (i.e., 4, *t*(48) = 3.08, *p* < .01). Also important, at the same time the other groups were perceived as significantly *less* meaningful than the midpoint of the scale, *ts*(48) > 3.09, *ps* < .01.

**Table 2:** Study 2 and Study 3 pretests. Average meaningfulness scores as a function of Type of Study (Study 2 and Study 3) and Type of Group. Standard deviations are in parenthesis.

	Ingroup	Bus stop	Green	Bank
	*M* (*SD*)	*M* (*SD*)	*M* (*SD*)	*M* (*SD*)
Study 2 (Left-handed)	4.71 (1.62)_a_	3.24 (1.45)_b_	3.27 (1.66)_b_	2.89 (1.51)_b_
Study 3 (Psychology)	5.59 (1.04)_a_	2.77 (1.18)_b_	2.80 (1.39)_b_	2.49 (1.10)_b_

Study 2 Ingroup = left-handed (N = 49); Study 3 Ingroup = Psychology students (N = 50); Bus stop = people at a bus stop; Green = people who like the color Green; Bank = people in line at a bank. Means across each row that do not share the same subscript are significantly different from each other at p < .001

#### Participants

Given the difficulty in recruiting left-handed individuals (it is estimated that approximately only 10% of the population is left-handed), the sample included only 41 left-handed students at the University of Padova who volunteered to participate in this experiment. Given the small sample size, Type of Individual was manipulated within-person, which reduces the interindividual variability. However, one participant who did not respond to one third of the questionnaire was eliminated from the data analysis so that the final sample included only 40 participants. We ran a sensitivity power analysis to detect the minimal detectable effect (MDE) we could detect with the number of available participants: α = .05, 1 - β = .80, *N* = 40. Results indicated that the smallest effect we could detect was Cohen’s *f* = .39, which falls in the medium effect area [26].

#### Design and procedure

The procedure was virtually identical to Study 1 except that the target group was “left-handed people” and the experiment was conducted in a University laboratory. The experimental design was a mixed design with Type of Inference as the between-person factor (deduction *n* = 20; induction *n* = 20) and Type of Individual as the within-person variable. The order of ratings involving the two types of individual (self and other) was counterbalanced across participants and produced no effects. Finally, to test the role of ingroup identification participants were asked to respond to a four-item ingroup identification scale (*I identify with the group of left-handed people*, *I am proud to be left-handed*, *I feel strong ties with the group of left-handed people*, *For me it is important to be left-handed*; α = .85; adapted from [27]). Responses could range from 1 = *Not at all* to 7 = *Very much*.

### Results

#### Inferred similarity between group and individual

For each participant, we computed average difference scores (i.e., average d^2^) representing the average of the squared differences between given and inferred scores. Thus, lower scores indicate stronger inferred similarity between ingroup and individual. We performed a 2 X 2 ANOVA on the average within-person d^2^ scores with Type of Inference (induction or deduction) as the first (between-persons) factor and Type of Individual (self or other) as the second (within-person) factor. Consistent with predictions, a significant main effect of Type of Inference was found (*F*(1,38) = 4.71, *p* = .04, η^2^ = .11) with a clear preference to rely on deduction than induction (see Table 1). No other effects were found. To test for the role of ingroup identification a multiple regression was conducted on the difference between self and other d^2^ scores. Please notice that *n* = 5 participants did not complete the identification measure. Neither a main effect of identification (*p* > .47) nor any interaction with the other variables was found (*ps* > .39). Therefore we were able to include ingroup identification as a covariate in the repeated measures ANOVA: the effect of Type of Inference was still significant (*F*(1,32) = 10.46, *p* = .003, η^2^ = .25) and no other effects were found (*p*s > .13).

Overall, as predicted, participants preferred to rely on deduction than induction; this effect was independent of ingroup identification.

## Study 3

Since Study 1 and Study 2 relied on minority groups (i.e., Sorority women and left-handed people), the goal of Study 3 was to extend and strengthen Studies 1 and 2 findings to a meaningful non-minority group. Because Psychology students at the University of Padova represent one of the largest majors, and Psychology at the University of Padova ranks highly at the national level, they were chosen as the meaningful non-minority ingroup for Study 3. A pretest was also conducted to ascertain that Psychology students perceive the ingroup as a meaningful group. Again, we expect participants to rely more on deduction than induction.

### Method

#### Pre-test

The same meaningfulness survey described in Study 2 was also conducted on a sample of Psychology students (*N* = 50). Results showed that Psychology students perceived their ingroup as significantly *more* meaningful than the non-meaningful groups (see Table 2). Moreover, Psychology students perceived their ingroup significantly *more* meaningful than the midpoint of the scale (i.e., 4; *t*(49) = 10.74, *p* < .001).

#### Participants

We decided a priori to recruit 206 Psychology students at the University of Padova who volunteered to participate in the experiment. This decision was supported by a sensitivity power analysis, α = .05, 1 - β = .80, *N* = 206, which showed that the minimal detectable effect (MDE) we could detect with the available sample was Cohen’s *f* = .20, which falls in the small effect area [26].

#### Design and procedure

The design (between persons) and procedure were the same as those used in Study 1 except that the target group was “Psychology students” and it took place in a University laboratory. Thus, random assignment resulted in four groups of participants: induction/self *n* = 51; induction/other *n* = 52; deduction/self *n* = 52; deduction/other *n* = 51. It should be noted that technically in Italy there is no such a thing as a University major. Thus, Psychology students enroll in the School of Psychology and typically attend almost exclusively Psychology classes for the entire duration of the curriculum (3 to 5 years).

Moreover, at the end of the Experiment, participants were asked to complete a 7-point identification scale consisting of four items (*I feel part of the group of Psychology students*, *I am proud to be a student in Psychology*, *I identify with the group of Psychology students*, *It is important for me to be a Psychology student;* α = .81, adapted from [27]). At the very end participants were asked to fill-out the same self-typicality scale as in Study 1.

### Results

#### Inferred similarity between group and individual

A 2 X 2 ANOVA with Type of Inference (induction or deduction) as the first (between-persons) factor and Type of Individual (self or other) as the second (between-persons) factor, was performed on average within-person d^2^ scores. Consistent with predictions, a significant main effect of Type of Inference was found (*F*(1,202) = 21.60, *p* < .001, η^2^ = .09) with a clear preference to rely on deduction than induction (see Table 1). Moreover, a main effect of Type of individual was found, with smaller d^2^ scores for other (*M* = 3.62, *SD* = 2.03) than for self (*M* = 4.35, *SD* = 2.75; *F*(1,202) = 5.17, *p* = .02, η^2^ = .02). No interaction effect was found (*F* < 1). To test for the role of ingroup identification with the ingroup, this variable was included in the general linear model: Neither a main effect of ingroup identification (*p* > .22) nor any interaction with other variables was found (*p* > .36). Therefore we were able to include ingroup identification as a covariate in the ANOVA: both the effects of Type of Inference (*F*(1, 201) = 21.81, *p* < .001, η^2^ = .10) and Type of Individual (*F*(1, 201) = 5.10, *p* = .02, η^2^ = .02) remained significant and no other effects were found (*p* > .41). Similarly, when the average level of self-typicality with the ingroup was included, neither a main effect of self-typicality (*p* > .17) nor any interaction with other variables was found (*p* > .31). Therefore we were able to include self-typicality as a covariate in the ANOVA: both the effects of Type of Inference (*F*(1,201) = 21.97, *p* < .001, η^2^ = .10) and Type of Individual (*F*(1,201) = 5.42, *p* = .02, η^2^ = .02) remained significant and no other effects were found (*p* > .07).

Overall, participants relied more on deduction than induction and this effect was independent of whether the self or another ingroup member was involved in the inferential task. Moreover, these findings were independent of the extent to which participants identified with or perceived themselves typical of the ingroup.

#### Meta-analysis and cross-experimental validation

In Studies 1, 2 and 3, participants reported higher preference for deduction over induction processes. Following the procedure outlined by Riva, Brambilla & Vaes [28], we meta-analytically combined the results from the effect sizes reported in Studies 1, 2 and 3. The meta-analysis showed that the weight-combined *Z*-score for condition (induction vs. deduction) was statistically significant (*Z* = 5.06, *p* < .01). The effect size of this preference for deduction over induction in meaningful group settings was intermediate (*d* = .55, η^2^ = .07; [26]).

Moreover, we conducted a cross-experimental validation of the significant effects of Study 1 and Study 3. By using the cross-experimental validation procedure we were able to enhance the *N* (i.e., test our predictions by relying on a larger sample) and ascertain the independence of our results from the specific type of ingroup (i.e., sororities and psychology students). Participants sample of Study 1 and Study 3 were students. Also, and differently from Study 2 (which included Type of Individual as a within-person factor), the experiment was identical in Study 1 and Study 3. Hence, statistical cross-examination of these studies (1 and 3) was theoretically reliable (for a similar procedure, see [29,30]). In this analysis, Type of Study (Study 1 vs. Study 3) was used as a between-participants factor and Type of Individual (self vs. other) and Type of Inference (induction vs. deduction) were used as between-participants variables; the d^2^ was the dependent variable. The overall sample was *N* = 329 participants. The main effect of Type of Study was not significant *F*(1,328) = .28, *p* = .60, η^2^ *=* .001, and did not interact with any other variable *p* > .27, η^2^ *<* .003. Importantly, in line with results of studies 1, 2, and 3 separately, the main effect of Type of Inference was significant *F*(1,328) = 18.25, *p* = .001, η^2^ *=* .05, and indicated a stronger preference for deduction (*M* = 3.43, *SE* = .21) over induction (*M* = 4.70, *SE* = .21). In line with results of studies 1, 2, and 3 separately, Type of Inference by Type of Individual interaction was not significant *F*(1,328) = 1.07, *p* = .30, η^2^ *=* .003. Moreover, unlike Study 3, the main effect of Type of Individual was not significant *F*(1,328) = 3.68, *p* = .06, η^2^ *=* .01. In sum, even when using a larger *N*, findings indicated a stronger preference for deduction over induction both for the self and the ingroup member in case of meaningful groups, which is not moderated by Type of Individual. Importantly, this pattern of results was independent of the type of group, i.e., sorority women or psychology students. Moreover, in Study 4 we further collected additional evidence on the preference for deduction over induction in meaningful group contexts, thus corroborating the results of the meta-analysis and the cross-experimental validation.

### Discussion

The goal of first three studies was to assess the inference process in the context of meaningful ingroups. Consistent with predictions, Sorority women (Study 1), left-handed people (Study 2), and Psychology students (Study 3) were more willing to attribute ingroup characteristics to the self or another ingroup member (i.e., self-stereotyping and ingroup-stereotyping) than to attribute self or other characteristics to the ingroup (i.e., self-anchoring and other-anchoring). The present findings concerning the preference for deduction over induction were corroborated by the cross-experimental validation including Studies 1 and 3. Moreover, the meta-analysis of Studies 1, 2, and 3 showed an intermediate effect size, suggesting that the preference for deduction over induction in meaningful group contexts is not trivial. Overall, meaningful groups clearly guide the preference for deduction over induction. Moreover, the Induction Deduction Paradigm allowed us to demonstrate that this preference occurs not only when the self is present in the inference process (i.e., self-stereotyping), but also when another ingroup member is involved (i.e., ingroup-stereotyping).

To extend and strengthen findings from Studies 1, 2, and 3, a fourth experiment was designed to provide a direct test of the hypothesis that the inference process is moderated by the perceived meaningfulness of the ingroup.

## Study 4

In this study we aim at providing stringent evidence that the level of the perceived ingroup meaningfulness moderates the inference process. Because the level of meaningfulness of Zodiac signs to the self varies across individuals depending on how strongly they perceive the Zodiac system as psychologically real and diagnostic of individual characteristics, we measured participants’ level of attributed meaningfulness to the Zodiac system and tested the prediction that such degree of meaningfulness would moderate their tendency to rely on self-stereotyping. Therefore, extending the self-stereotyping findings shown by Sorority women, left-handed people, and Psychology students, we expect self-stereotyping to increase with higher levels of attributed meaningfulness to the Zodiac system. In addition, based on Studies 1, 2, and 3 findings, we expect that for higher levels of attributed meaningfulness, deduction would be generally preferred over induction also when another individual is the target. By contrast, for decreased levels of attributed meaningfulness, we expect a different pattern of inference depending on the target, i.e., a replication of previous results in minimal group settings by Cadinu and Rothbart [8]. Therefore, for low levels of meaningfulness induction should be preferred over deduction when the self is the target with the reverse pattern expected when the other individual is the target. Furthermore, we explored the possibility that knowledge about Zodiac signs would be a moderator of the inference process. This exploration is based on previous research on the important role of group clarity in predicting which inference process leads to identification and the reasoning that group knowledge might be considered a proxy for group clarity [14] as lower knowledge concerning zodiac groups likely signals lower clarity on the contents of these groups.

It is worth noticing that treating the Zodiac as a minimal group is consistent with previous findings on inter-judge agreement regarding ingroup characteristics. Assuming that inter-judge agreement on the characteristics of a group reflects the degree of "reality" of a group, it should increase from minimal to meaningful groups. There is moderate consensus among University students about the personality characteristic of their University ingroup (inter-judge agreement *r*(350) = .20, *p* < .001). A significant but lower level of consensus was found among people belonging to the same Zodiac sign (inter-judge agreement *r*(350) = .13, *p* < .01). When consensus on the characteristics of the ingroup was computed on the basis of random sets of participants belonging to different Zodiac signs, the inter-judge agreement dropped significantly to *r*(350) = .06 (n.s.). These results are consistent with the idea that there is some small (non-zero) degree of agreement in characterizing one's own Zodiac sign.

Overall, in Study 4 we test the prediction that the inference process is moderated by the level of the attributed meaningfulness to the Zodiac ingroup resulting in a three-way interaction among type of inference, type of individual, and level of attributed meaningfulness to zodiac signs.

### Method

#### Participants

Participants were 136 undergraduates (74 women, 60 men and 2 of unknown gender) who volunteered to participate in this experiment in partial fulfillment of course requirements. As for Studies 1, 2, and 3, we ran a sensitivity analysis to test the minimal detectable effect we could find with the collected number of participants, which provided the following results: α = .05, 1 –β = .80, *N* = 136, *f*^*2*^ = .11. Therefore, the sample size was powered enough to detect at least a small/intermediate effect [26].

#### Procedure

The design (between persons) and the procedure were the same as in Experiment 1 except that the target group was “people belonging to your Zodiac sign” and it took place in a University laboratory. Thus, random assignment resulted in four groups of participants: induction/self *n* = 31; induction/other *n* = 36; deduction/self *n* = 35; deduction/other *n* = 34. Moreover, at the end of the experiment, participants were asked to make additional judgments: Rating of their knowledge of the Zodiac system on a 6-point scale that ranged from *Until this study I was unaware of the Zodiac system involving Zodiac signs* (1) to *I have extensive knowledge of the system and of the characteristics of all signs* (6). Most importantly for our purpose, we operationalized perceived meaningfulness of the Zodiac system as the extent to which participants attributed to the Zodiac sign system the status of ‘real existence’. This allowed us to measure individual differences in term of attributed meaningfulness to the Zodiac system. Specifically, participants provided their rating on a 7-point scale that ranged from *I don't accept it at all* (1) to *I consider it to be true* (7) in response to the question: *To what extent do you personally accept*, *or believe in*, *the assumption that there is a correspondence between the sign under which a person is born and the nature of that individual personality*? (*M* = 3.82, *SD* = 1.61). Finally, due to a procedure error, only 79 out of 136 participants responded to the self-typicality scale. Thus, self-typicality was not included as a moderator in data analysis and will not be further discussed.

### Results

#### Inferred similarity between group and individual

For each participant, we computed average difference scores (i.e., average d^2^) representing the average of squared differences between given and inferred scores. The effect of participants' level of Attributed Meaningfulness to the Zodiac system on the inference task was assessed in the context of a moderated model using PROCESS (model n° 3; [31]) with 5000 bootstrapping samples. Specifically, we tested the effect of Type of Inference (induction = 0, deduction = 1) on participants’ d^2^ based on the conditional effects of Attributed Meaningfulness to the Zodiac system (continuous, centered) and Type of Individual (other = 0, self = 1) as moderators. As shown in Table 3, the three-way interaction Type of Inference x Type of Individual x Attributed Meaningfulness significantly increased the amount of the explained variance (*ΔR*^*2*^ = .03, *R*^*2*^ = .10, *p* = .03) even though the overall model fell short of significance, *F*(7,127) = 1.95, *p* = .07. Specifically, in support of the moderation hypothesis, the two-way interaction between Type of Individual and Type of Inference (*t* = 2.28, *p* = .02) was further qualified by a three-way interaction between Type of Individual, Type of Inference and Attributed Meaningfulness (*t* = 2.22, *p* = .03; 95% LLCI = -3.28, ULCI = -.19).

**Table 3:** Study 4. Moderated multiple regression with Type of Inference (0 = Induction, 1 = Deduction), Type of Individual (0 = Other, 1 = Self) and level of Attributed Meaningfulness of the Zodiac system (continuous, centered) and all interactions predicting inferred similarity between given and inferred scores (d^2^ scores).

	*b*	*SE b*	*R*^*2*^	*ΔR*^*2*^	*F (dfs)*
Model			.10	.10	1.95 (7,127)^†^
Intercept	4.88**	.61			
Type of Inference	-1.66	.89			
Type of Individual	-1.16	.90			
Meaningfulness	-.59	.38			
Inference X Individual	2.89*	1.27			
Inference X Meaningfulness	.63	.53			
Individual X Meaningfulness	.64	.52			
Inference X Individual X Meaningfulness	-1.73*	.78		.03	4.91 (1,127)*

^†^p = .07

*p < .05

**p < .01

**Fig 1:**
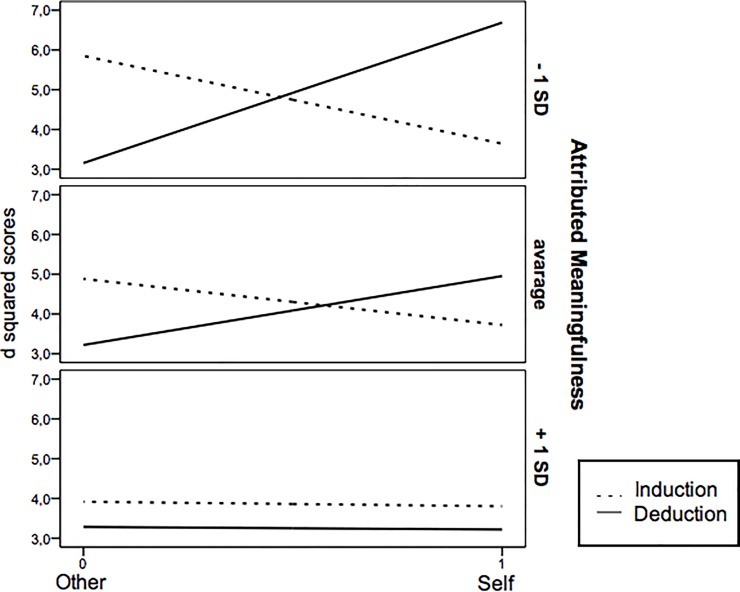
Study 4. Effect of type of inference (induction vs. deduction) on participants’ d^2^ scores based of the conditional effect of Attributed Meaningfulness (-1SD, average, +1SD) and Type of Individual (self vs. other) as moderators.

Fig 1 shows the pattern of results for different levels of Attributed Meaningfulness to the Zodiac system (- 1 *SD*, Average, +1 *SD*). As predicted, for low levels of Attributed Meaningfulness (- 1 *SD*; see highest panel of Fig 1) the Type of Inference x Type of Individual interaction was significant (*b =* 5.74, *SE =* 1.80, *t* = 3.18, *p* < .01; 95% LLCI = 2.17, ULCI = 9.31); specifically, for low levels of Attributed Meaningfulness the conditional effect of Type of Inference on d^2^ was significant both for the self (*b =* 3.05, *SE =* 1.32, *t* = 2.31, *p* = .02; 95% LLCI = .44, ULCI = 5.65) and other Type of Individual (*b = -*2.69, *SE =* 1.23, *t* = -2.18, *p* = .03; 95% LLCI = -5.14, ULCI = -.25). Consistent with the pattern of results in minimal group settings [8], these findings clearly show that participants engage in self-anchoring by inferring more similarity (lower d^2^ scores) in the induction over deduction condition when the self is involved whereas they infer more similarity (lower d^2^ scores) under deduction over induction when the other ingroup member is involved, i.e., ingroup-stereotyping (see plotted values < 1 *SD* in the top panel of Fig 1). On the contrary, for high levels of Attributed Meaningfulness (> 1 *SD;* lowest panel of Fig 1), as predicted the conditional effect of Type of Inference x Type of Individual interaction was not significant (*t* < .03; *p* > .97) and the conditional effect of Type of Inference was not significant either for the self or the other (*ts* < .50; *ps* > .65). Importantly, plotted values in the lowest panel of Fig 1 indicate a main effect of Type of Inference specifically for those participants with high levels of Attributed Meaningfulness, who inferred higher similarity (i.e., lower d^2^) in the deduction over induction condition regardless of Type of Individual (self or other). To support this claim, we conducted the same moderation model as above (PROCESS model n° 3 with 5000 bootstrapping samples; Hayes [31]) only on those participants who displayed the highest levels of Attributed Meaningfulness to the Zodiac system (i.e., above 1.48; approximately 33% participants, *n* = 44). As predicted, only the main effect of Type of Inference was significant (*b* = -10.33, *SE =* 4.58; *t* = -2.26, *p* = .03; 95% LLCI = -19.62, ULCI = -1.05), suggesting that, for the highest levels of Attributed Meaningfulness, inferred similarity was stronger in the deduction than induction condition. Hence, confirming findings of Studies 1, 2, and 3, this pattern was not moderated by the Type of Individual: For participants who appraised zodiac groups as most meaningful, deduction was stronger than induction regardless of whether the self or the other ingroup member was involved in the inferential task.

*Zodiac knowledge*. The level of knowledge about the Zodiac system was analyzed as another possible moderator. The correlation between Knowledge and level of Attributed Meaningfulness to the Zodiac system was *r*(133) = .48, *p* < .001. Similarly to Attributed Meaningfulness, participants' level of Zodiac Knowledge was analyzed in the context of a moderated model using PROCESS (model n° 3, 5000 bootstrapping samples; [31]). Specifically, we tested the effect of Type of Inference (induction = 0, deduction = 1) on participants’ d^2^ based on the conditional effects of Zodiac Knowledge (continuous, centered), and Type of Individual (other = 0, self = 1) as moderators. The overall model was not significant, *F*(7,126) = .98, *p* = .45, *R*^*2*^ = .06. Moreover, the two-way interaction Type of Inference x Type of Individual (*b =* 2.91, *t =* 2.21, *p =* .03) was not qualified by a three-way interaction Type of Inference x Type of Individual x Zodiac Knowledge (*b = -*.25, *t =* -.19, *p =* .85). No other effects were found (*ps >* .08). Therefore, we were able to include Zodiac Knowledge as a covariate in a comprehensive model testing the effect of Type of Inference (induction = 0, deduction = 1) on participants’ d^2^ based on the conditional effects of Attributed Meaningfulness to the Zodiac system (continuous, centered) and Type of Individual (other = 0, self = 1). Specifically, both the two-way interaction between Type of Individual and Type of Inference (*b* = 2.89, *t* = 2.17, *p* = .03) and the three-way interaction between Type of Individual, Type of Inference and Attributed Meaningfulness (*b* = - 1.75, *t* = 2.16, *p* = .03; 95% LLCI = -3.35, ULCI = -.15) remained significant when including Zodiac Knowledge as a covariate. No other effects were found (*p >* .11).

### Discussion

In Study 4, we used participants' Zodiac sign as the ingroup to test the hypothesis that different levels of group meaningfulness would predict different inference strategies. Results indicated that inference strategies radically changed as a function of the level of attributed meaningfulness to the Zodiac system and not as a function of the knowledge (i.e., clarity) about the Zodiac system. As hypothesized participants with high levels of attributed meaningfulness to the Zodiac system were more willing to generalize ingroup information to the individual, and this pattern of deduction was similar for the self and the other type of individual, thus leading to self-stereotyping and ingroup-stereotyping. Importantly, this pattern of results mirrors the findings of Study 1, Study 2, and Study 3, attesting that processing the ingroup as meaningful leads participants to rely on ingroup information to shape the representation of its individual members. By contrast, lower levels of attributed meaningfulness to the ingroup were associated with a radically different pattern of inferences involving the self and the other individual. Specifically, participants who attributed low meaningfulness to the ingroup were more willing to generalize self-related information to the ingroup (self-anchoring vs. self-stereotyping) and to generalize ingroup information to the other ingroup individual (ingroup-stereotyping vs. other-anchoring). Importantly, the last pattern of results is consistent with results in minimal group settings [8] demonstrating that artificial, i.e., non-meaningful, ingroups lead to self-anchoring and ingroup-stereotyping. In sum, these results support the overall prediction of the present study that the meaningfulness of the ingroup is a crucial variable in predicting the causal direction of the inference process. However, given the size of the experimental sample, and the expectations concerning the three-way interactions, future studies could address the same research question by relying on a larger sample size.

## General discussion

The current research program showed three important sets of findings. The first regards the direct demonstration of the causal direction between self and ingroup in meaningful social categories. The reliance on the methodologically stringent Induction Deduction Paradigm [8] allowed us to clearly determine the causal direction of individual-ingroup inference by providing feedback on the individual or the ingroup along unfamiliar psychological dimensions on which participants could have no previous knowledge. As predicted, our findings (Studies 1, 2, and 3) showed that people are more willing to attribute ingroup characteristics to the self (self-stereotyping) than self-characteristics to the ingroup (self-anchoring) when dealing with meaningful ingroups, such as those represented by social categories (i.e., sorority women, left-handed people, Psychology students). In sum, we provided the first direct demonstration that self-stereotyping prevails over self-anchoring in meaningful group contexts. These results, which were obtained with meaningful groups, are consistent with previous research derived from Self-Categorization Theory on self-stereotyping and self-categorization, which shows that participants see themselves as more similar to the ingroup when the ingroup is particularly meaningful, for example in minority vs. majority conditions [11,22,32–35], and also when the ingroup is associated with positive versus negative characteristics [36].

The second novel set of findings demonstrated the direction of influence between ingroup and individual when another ingroup member rather than the self is involved in the inference task. As predicted, for sorority women, left-handed people, and Psychology students ingroup-stereotyping (i.e. ingroup-to-individual deduction) prevailed over other-anchoring (i.e., individual-to-ingroup induction). Thus, both self-stereotyping and ingroup-stereotyping prevailed over self-anchoring and other-anchoring in the context of meaningful social groups. These results are consistent with Self-Categorization Theory premise [23] that when ingroups are perceived as meaningful, individuals are more willing to incorporate part of their ingroup representation both into their self-perception and into the perception of another ingroup member, that is to shift from personal to social identity via the process of self-stereotyping and ingroup-stereotyping [11,22].

The third and most convincing set of findings regards the experimental test of the novel hypothesis that the inferential pattern (induction vs. deduction) is moderated by the degree of perceived meaningfulness of the ingroup. In line with Studies 1, 2, and 3, results in Study 4 showed that the more participants attributed meaningfulness to the Zodiac system, the higher their willingness to engage in self-stereotyping (vs. self-anchoring) and ingroup-stereotyping (vs. other-anchoring). By contrast, when the ingroup was processed as not psychologically real but stemming from a fictitious, unreasonable criterion for the categorization of people, a dramatically different pattern of results occurred. In line with the self-anchoring model [8], when participants attributed low meaningfulness to their Zodiac ingroup, they used the self as a reliable anchor and thus preferred to generalize novel self-information to the ingroup than to generalize ingroup information to the self (i.e., self-anchoring vs. self-stereotyping). On the contrary, when another ingroup member was involved in the inference task, individuals did not possess a reliable anchor to base their inductive inference. In this case they likely considered a single information based on an in-group member as less reliable than information based on the average of multiple observations concerning the whole group, and thus preferred to generalize group information to the individual rather than generalize information on a single individual to the group as a whole (i.e., ingroup-stereotyping vs. other-anchoring). Hence, it was specifically self-related information, and not information related to any ingroup individual, that was taken as the standard to anchor the representation of the ingroup. Notice that these results strongly replicate and extend former results previously found only in minimal group settings [8]. Overall, Study 4 represents the strongest test of meaningfulness in shaping inferential strategies based on deduction or induction.

Although meaningfulness was the focal moderator of the inferential pattern of the results, given the crucial role of ingroup identification, self-typicality and group clarity (i.e., knowledge) in shaping individual-group dynamics (e.g., [13,14,22,25]) we also explored the possibility that these variables would moderate the inference pattern. Results showed that the preference for deduction over induction was not moderated by any of these variables. Hence, inferential processes in meaningful contexts appear to be independent of these constructs. Conjecturally, this pattern of results may be strongly dependent on the current experimental setting and generalization of these findings to other settings should be made with caution. Indeed, differently from studies in which individual-group similarity was assessed on familiar traits, and in which participants held prior knowledge on the ingroup or the self, participants in the current studies were provided with information on unfamiliar dimensions when making inference about either the individual or the ingroup. One possibility is that when prior knowledge on the self/ingroup is available and when participants reason on information that is familiar, group knowledge or clarity may become relevant, participants may rely on the perceived level of fit between the representation of the self and the ingroup representation (i.e., self-typicality), and/or rely on the importance to the self that is attributed to the ingroup (i.e., ingroup identification) to guide the inferential process. In contrast, when information is based on unfamiliar dimensions, as in the present study, prior group knowledge and group clarity are low, and how strongly participants identify with or consider the self as typical of the ingroup may not be very relevant in guiding the inference process. In such a context, it might be plausible that participants use the rules of logic that it is better to base inferences on the average of multiple data points (i.e., information on a meaningful ingroup) and then generalize group average information to the individual (i.e., self-stereotyping and ingroup-stereotyping) than to generalize a single data-point on one individual (self or other ingroup member) to the whole group. Given the speculative nature of these arguments, future studies may compare the potential moderating role of ingroup identification, self-typicality, and group-clarity when either familiar or unfamiliar information is used as the basis for inference involving the individual and the ingroup.

### Future directions

#### Manipulation of meaningfulness

One limitation of the present study is that meaningfulness was not experimentally manipulated. To further support the role of meaningfulness, in a future study participants could be told that their membership (e.g., people with spatial ability predominance) is determined either by genetic or random variations in the population. Because the former group would presumably be considered more meaningful than the latter group, we should expect opposite inferential patterns with the Induction Deduction Paradigm in the two groups of participants.

#### Role of other moderators

Although Study 4 pattern of results clearly demonstrates that the inference pattern is moderated by the attributed meaningfulness to the group independent of group knowledge (i.e., group clarity), in this experiment the role of other moderators has not been tested. Therefore, future research might also measure for example ingroup identification and test its role as a moderator of the inference pattern together with attributed meaningfulness. Such a test might show the relative strength of these moderators, thus contributing to further understand how people construe the representation of the ingroup and its members.

#### Future research designs

Another limitation of the present study concerns the lack of a control condition or a baseline assessment of the strength of the inferential process (induction vs. deduction) involving the individual and an uncategorized group of people. For instance, and with respect to Studies 1–3, providing uncategorized participants with feedback on their performance, and asking them to infer the performance of a group of other uncategorized individuals, would allow researchers to assess participants’ inductive inference when no ingroup is involved. Similarly, giving feedback concerning the performance of a group of other uncategorized individuals and asking the participant to infer her/his own performance, would allow researchers to measure participants’ deductive inference when no clear definition of the self as an ingroup member has been made salient. This would allow to compare not only self-anchoring against self-stereotyping and other-anchoring against ingroup-stereotyping, but also to compare each of these processes against a baseline condition.

### Implications

#### Socialization processes

The present findings have implications for the processes of socialization in groups or organizations. In these contexts, the ingroup may become progressively more meaningful to the individual as time goes on, thus reinforcing the preference for self- and ingroup-stereotyping over self- and other-anchoring. This speculation is in line with results by van Veelen et al. [37], showing that newcomers in a group rely more on self-anchoring whereas well-established members rely more on self-stereotyping to instantiate ingroup identification [38].

#### Individual differences

The present results may also be related to the study of individual characteristics. For example, one can speculate that individuals who are high on narcissism may not take into strong consideration the meaningfulness of the ingroup and rely more on individual information in the inference process, thus preferring self-anchoring over self-stereotyping. This conjecture is in line with research showing that narcissism is positively related to an independent self-construal and negatively related to a group-focused self-construal [39].

#### Interventions

The present study has also important intervention implications. Results revealed that for meaningful groups, individuals derive self-representation from the ingroup representation. Clearly, the ingroup-to-self deductive processes might favor self-esteem maintenance and enhancement, as detailed by Social Identity Theory [40]. However, the group-to-self deduction might also have detrimental consequences, as in the case of stereotyped groups (e.g., women, lesbians). For instance, gender stereotypes about math-skills may account for women’s underperformance in math-related domains (e.g., [41]), and negative stereotypes about lesbian women can be associated with lower levels of self-esteem in this group (e.g., [42]). Importantly, our findings suggest that to the extent that individuals do not attribute meaningfulness to the intergroup distinction, the power of the ingroup representation to shape the representation of the self becomes weaker. Hence, these results suggest that dismissing the meaningfulness of the group distinction, for example through educational interventions aimed at invalidating gender differences or revising stereotypes about women, gay and lesbian individuals, might reduce the impact of negative group characteristics to the self, and possibly free up individuals’ self-perception, skills, attitudes, and behaviors from intergroup dynamics.

## Supporting information

S1 Material Cognitive tasks 1 & 4. (PDF)

S2 Material Cognitive tasks 2 & 3. (PDF)

S3 Material Inference task. (DOCX)

S1 Dataset (SAV)

S2 Dataset (SAV)

S3 Dataset (SAV)

S4 Dataset (SAV)
