# Supplementary figures and images for: Group meaningfulness and the causal direction of influence between the ingroup and the self or another individual: Evidence from the Induction-Deduction Paradigm
Source: PLoS One. 2020 Mar 10;15(3):e0229321. doi: 10.1371/journal.pone.0229321 (PMC7064197; doi:10.1371/journal.pone.0229321)

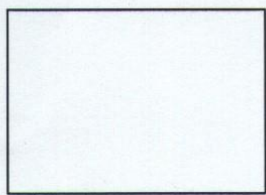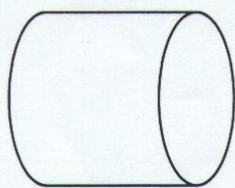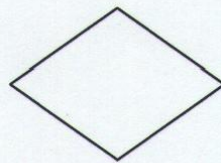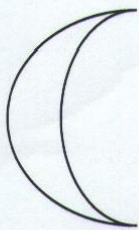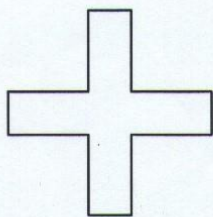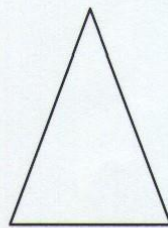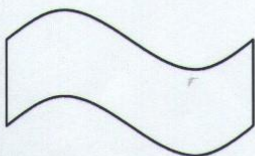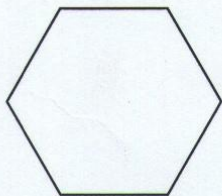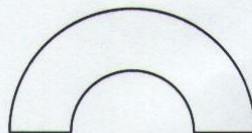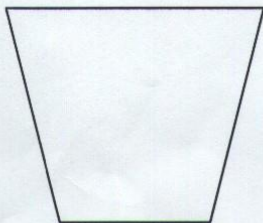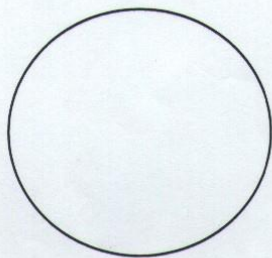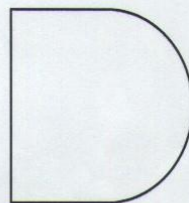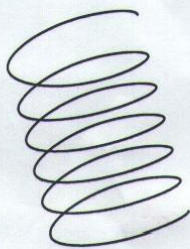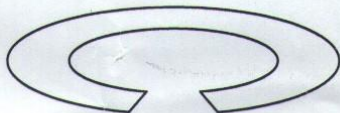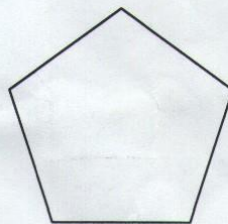

Supplement: S1 Material — (PDF) [file pone.0229321.s001.pdf]
