# Supplementary material for: Group meaningfulness and the causal direction of influence between the ingroup and the self or another individual: Evidence from the Induction-Deduction Paradigm
Source: PLoS One. 2020 Mar 10;15(3):e0229321. doi: 10.1371/journal.pone.0229321 (PMC7064197; doi:10.1371/journal.pone.0229321)
Supplement: S3 Material — (DOCX) [file pone.0229321.s003.docx]

PSYCHOLOGICAL SCALE: CLUSTERING IN DIGITAL SPAN RECALL

The tendency to group items together when a person is presented with a list of numbers and asked to memorize it. High scores on the clustering scale indicate good performance in recall and recognition tasks.

How your **Sorority House** scored:

| Very uncharacteristic | Somewhat uncharacteristic | Neither characteristic nor uncharacteristic | Somewhat characteristic | Very characteristic |
| --- | --- | --- | --- | --- |
| ǀ | ǀ | ǀ | ǀ | ǀ |

1---------2----------3---------4----------5----------6----------7----------8---------9

How do you think **you personally** would score? (circle one)

| Very uncharacteristic | Somewhat uncharacteristic | Neither characteristic nor uncharacteristic | Somewhat characteristic | Very characteristic |
| --- | --- | --- | --- | --- |
| ǀ | ǀ | ǀ | ǀ | ǀ |

1---------2----------3---------4----------5----------6----------7----------8---------9

Rate how socially desirable it is to have a high score on **CLUSTERING IN DIGITAL SPAN RECALL**

| Very undesirable | Somewhat undesirable | Neither desirable nor undesirable | Somewhat desirable | Very desirable |
| --- | --- | --- | --- | --- |
| ǀ | ǀ | ǀ | ǀ | ǀ |

1---------2----------3---------4----------5----------6----------7----------8---------9

PSYCHOLOGICAL SCALE: PARALLEL INFORMATION PROCESSING

The tendency to process various parts or aspects of incoming information in parallel with other parts or aspect of information. High scores on the Pararrel Processing Scale indicate good comprehension process.

How your **Sorority House** scored:

| Very uncharacteristic | Somewhat uncharacteristic | Neither characteristic nor uncharacteristic | Somewhat characteristic | Very characteristic |
| --- | --- | --- | --- | --- |
| ǀ | ǀ | ǀ | ǀ | ǀ |

1---------2----------3---------4----------5----------6----------7----------8---------9

How do you think **you personally** would score? (circle one)

| Very uncharacteristic | Somewhat uncharacteristic | Neither characteristic nor uncharacteristic | Somewhat characteristic | Very characteristic |
| --- | --- | --- | --- | --- |
| ǀ | ǀ | ǀ | ǀ | ǀ |

1---------2----------3---------4----------5----------6----------7----------8---------9

Rate how socially desirable it is to have a high score on **PARALLEL INFORMATION PROCESSING**

| Very undesirable | Somewhat undesirable | Neither desirable nor undesirable | Somewhat desirable | Very desirable |
| --- | --- | --- | --- | --- |
| ǀ | ǀ | ǀ | ǀ | ǀ |

1---------2----------3---------4----------5----------6----------7----------8---------9

PSYCHOLOGICAL SCALE: GLOBAL ORIENTATION IN DESIGN CONSTRUCTION

The tendency to start the construction of a design in a global rather than in an analytic way. High scores on the Global Orientation Scale indicate an efficien construction strategy.

How your **Sorority House** scored:

| Very uncharacteristic | Somewhat uncharacteristic | Neither characteristic nor uncharacteristic | Somewhat characteristic | Very characteristic |
| --- | --- | --- | --- | --- |
| ǀ | ǀ | ǀ | ǀ | ǀ |

1---------2----------3---------4----------5----------6----------7----------8---------9

How do you think **you personally** would score? (circle one)

| Very uncharacteristic | Somewhat uncharacteristic | Neither characteristic nor uncharacteristic | Somewhat characteristic | Very characteristic |
| --- | --- | --- | --- | --- |
| ǀ | ǀ | ǀ | ǀ | ǀ |

1---------2----------3---------4----------5----------6----------7----------8---------9

Rate how socially desirable it is to have a high score on **GLOBAL ORIENTATION IN DESIGN CONSTRUCTION**

| Very undesirable | Somewhat undesirable | Neither desirable nor undesirable | Somewhat desirable | Very desirable |
| --- | --- | --- | --- | --- |
| ǀ | ǀ | ǀ | ǀ | ǀ |

1---------2----------3---------4----------5----------6----------7----------8---------9

PSYCHOLOGICAL SCALE: FIELD ORIENTATION IN PROBLEM-SOLVING

The tendency to rely on relational information present in the problem context. High scores on the Field Orientation Scale indicate good problem-solving strategy.

How your **Sorority House** scored:

| Very uncharacteristic | Somewhat uncharacteristic | Neither characteristic nor uncharacteristic | Somewhat characteristic | Very characteristic |
| --- | --- | --- | --- | --- |
| ǀ | ǀ | ǀ | ǀ | ǀ |

1---------2----------3---------4----------5----------6----------7----------8---------9

How do you think **you personally** would score? (circle one)

| Very uncharacteristic | Somewhat uncharacteristic | Neither characteristic nor uncharacteristic | Somewhat characteristic | Very characteristic |
| --- | --- | --- | --- | --- |
| ǀ | ǀ | ǀ | ǀ | ǀ |

1---------2----------3---------4----------5----------6----------7----------8---------9

Rate how socially desirable it is to have a high score on **FIELD ORIENTATION IN PROBLEM-SOLVING**

| Very undesirable | Somewhat undesirable | Neither desirable nor undesirable | Somewhat desirable | Very desirable |
| --- | --- | --- | --- | --- |
| ǀ | ǀ | ǀ | ǀ | ǀ |

1---------2----------3---------4----------5----------6----------7----------8---------9

PSYCHOLOGICAL SCALE: LATERALIZATION OF BRAIN FUNCTIONS IN LANGUAGE COMPREHENSION

The tendency for brain funcions to be highly distributed within one of the two cerebral hemispheres. High scores on the Lateralization Scale indicate an efficient and quick comprehension process.

How your **Sorority House** scored:

| Very uncharacteristic | Somewhat uncharacteristic | Neither characteristic nor uncharacteristic | Somewhat characteristic | Very characteristic |
| --- | --- | --- | --- | --- |
| ǀ | ǀ | ǀ | ǀ | ǀ |

1---------2----------3---------4----------5----------6----------7----------8---------9

How do you think **you personally** would score? (circle one)

| Very uncharacteristic | Somewhat uncharacteristic | Neither characteristic nor uncharacteristic | Somewhat characteristic | Very characteristic |
| --- | --- | --- | --- | --- |
| ǀ | ǀ | ǀ | ǀ | ǀ |

1---------2----------3---------4----------5----------6----------7----------8---------9

Rate how socially desirable it is to have a high score on **LATERALIZATION OF BRAIN FUNCIONS IN LANGUAGE COMPREHENSION**

| Very undesirable | Somewhat undesirable | Neither desirable nor undesirable | Somewhat desirable | Very desirable |
| --- | --- | --- | --- | --- |
| ǀ | ǀ | ǀ | ǀ | ǀ |

1---------2----------3---------4----------5----------6----------7----------8---------9

PSYCHOLOGICAL SCALE: MODALITY DOMINANCE IN SYNESTESIC PERCEPTION

Refers to the simultaneous perception of different sensory modalities. High scores on the Modality Dominance Scale indicate an efficient perception process because the correct distincion between the two sensory modallities is obtained.

How your **Sorority House** scored:

| Very uncharacteristic | Somewhat uncharacteristic | Neither characteristic nor uncharacteristic | Somewhat characteristic | Very characteristic |
| --- | --- | --- | --- | --- |
| ǀ | ǀ | ǀ | ǀ | ǀ |

1---------2----------3---------4----------5----------6----------7----------8---------9

How do you think **you personally** would score? (circle one)

| Very uncharacteristic | Somewhat uncharacteristic | Neither characteristic nor uncharacteristic | Somewhat characteristic | Very characteristic |
| --- | --- | --- | --- | --- |
| ǀ | ǀ | ǀ | ǀ | ǀ |

1---------2----------3---------4----------5----------6----------7----------8---------9

Rate how socially desirable it is to have a high score on **MODALITY DOMINANCE IN SYNESTESIC PERCEPTION**

| Very undesirable | Somewhat undesirable | Neither desirable nor undesirable | Somewhat desirable | Very desirable |
| --- | --- | --- | --- | --- |
| ǀ | ǀ | ǀ | ǀ | ǀ |

1---------2----------3---------4----------5----------6----------7----------8---------9
